# Deltex1 Polymorphisms Are Associated with Hepatitis B Vaccination Non-Response in Southwest China

**DOI:** 10.1371/journal.pone.0149199

**Published:** 2016-02-19

**Authors:** Bin Xie, Penghui Zhang, Menggang Liu, Wei Zeng, Juntao Yang, Hongming Liu

**Affiliations:** 1 Department of Hepatobiliary Surgery, Daping Hospital & Institute of Surgery Research, Third Military Medical University, Chongqing, China; 2 Department of Clinical Laboratory Medicine, Children’s Hospital of Chongqing Medical University, Chongqing, China; Academia Sinica, TAIWAN

## Abstract

**Background:**

Hepatitis B vaccination is the most important tool available for preventing hepatitis B virus (HBV) infection and reducing the prevalence of infection. However, epidemiological studies have demonstrated that morethan 5% of patients exhibit a non- or hypo-response to the HBV vaccine. Genetic variations associated with T cell immunity contribute to the immune response to HBV vaccination. The deltex 1 (DTX1) gene is involved in T cell anergy, which may also be associated with the immune response to the HBV vaccination.

**Methods:**

We detected 10 single nucleotide polymorphisms (SNPs) in or around the DTX1 gene in 601 infants out of a population from Southwest China, including 299 high responders(HRs; HBsAb > 100 mIU/mL) and 302 non-responders (NRs; HBsAb < 10 mIU/mL). An additional validation study was performed, comprising 230 adult patients(135 HRs and 95 NRs) from Southwest China.

**Results:**

This study found that the minor allele ‘G’ of rs2384077 (adjusted *p* = 2.63E^-04^,) and the minor allele ‘C’ of rs10744794 (adjusted *p* = 3.69E^-04^) in the first intron of the DTX1 gene were remarkably associated with the immune response to HBV vaccination in both infant and adult populations. Moreover, a subsequent analysis indicated that haplotypes (A-T, G-C) of the two SNPs were significantly associated with the immune response to HBV vaccination.

**Conclusions:**

Two SNPs (rs2384077 and rs10744794) in an intron of DTX1 and the linkage disequilibrium (LD) block are significantly associated with the immune response to HBV vaccination. The functional element annotation of the LD block between the two SNPs contains four transcriptional regulatory elements. The results suggest that these two SNPs may be involved in the immune response to HBV vaccination.

## Introduction

Hepatitis B virus (HBV) infection is a global problem that tremendously impacts affected individuals’ health as well as society in general. It has been estimated that more than two billion people suffer from HBV infection, andapproximately350 million chronically infected people have a high mortality risk due to cirrhosis of the liver and liver cancer [1]. HBV vaccination is the most important tool available for preventing HBV infection and decreasing its prevalence[2, 3]; however, epidemiological studies have demonstrated that approximately 5%-10% of patients exhibit a non- or hypo-response that results in vaccine failure [4]. Therefore, uncovering the mechanism underlying this non- or hypo-response and finding solutions for vaccine failure are important medical issues.

Studies have demonstrated that HBV infection appears in family aggregations, which may be caused by both environmental and genetic factors. A non- or hypo-response to the HBV vaccine is often the result of several endogenous and exogenous factors, including psychological stress, nutrition, smoking, alcohol consumption, co-infection (along with HIV infection), gender, age, body weight, race, and the type and quantity of vaccine administered [5–7]. These factors can affect the immune response after vaccination by impacting the function of the immune system. Numerous studies have confirmed that the body’s response after vaccination is closely related to the genetic background of the immune system [8, 9]. For example, the deletion of genes in human leukocyte antigen complexes and deficits in antigen-presenting cell (APC) function were previously found to be associated with a non- or hypo-response to the HBV vaccine [10, 11]. In addition, single nucleotide polymorphisms (SNPs) in human leukocyte antigen (HLA), tumor necrosis factor (TNF), interferon (IFN), interleukin (IL)-1, IL-4, IL-10, IL-15 and corresponding genes are associated with HBV vaccine failure[12–19]. Nevertheless, most of the genes evaluated in prior studies are related to T cell activation, and there has been a lack of research on the functional impact of the affected genes.

The activation and proliferation of T cells are important steps in the immune response to HBV vaccination [20–22]. Dysfunction of helper T cells and special types of B cells due to underlying disease has been demonstrated to be related to a non- or hypo-response; the HBsAg of T cells are also related to vaccine failure. The immune response is largely controlled by T cells, which can recognize the HBsAg and regulate other immunocytes to maintain the production of HBsAb. One indication of successful HBV vaccination is continued HBsAb production[23].

Deltex1 (DTX1) is a single transmembrane protein that has been identified as a key factor in T cell immunity[24].Recently, Hsiao et al. demonstrated that DTX1 is a target of the transcription factor (TF) nuclear factor of activated T cells (NFAT). NFAT promotes T cell anergy and increased DTX1 expression, which may attenuate T cell activation [25]. We propose that DTX1 may play a role in the T cell response to HBV vaccination.

In this study, we detected 10 SNPs in or around the DTX1 gene in 601 infants from a population in Southwest China, including 299infants with successful vaccination (HBsAb > 100 mIU/mL) and 302 with vaccine failure (HBsAb<10 mIU/mL), using an improved multiplex ligation detection reaction (iMLDR) technique. A total of 230 adults from Southwest China were also examined in a validation study. Moreover, a haplotype analysis was performed to assess the functional relevance of the SNPs and to detect the DNA regions highly associated with the immune response to HBV vaccination.

## Materials and Methods

### Study population

This study was approved by the Experimental and Ethics Committees of Daping Hospital, Third Military Medical University, and the written informed consent was obtained from each participating subject or the subject’s guardian (for infants). Two independent Chinese Han populations were used in these studies. The first population was composed of infants recruited from the Chongqing Maternal and Child Care Service Centre. The following inclusion criteria were used for this population: (1) born full term; (2) body mass index (BMI) in the normal range; (3) HBsAg-negative status; and (4) an absence of underlying diseases, such as congenital heart disease, neonatal aspiration pneumonia, hypoxic-ischemic encephalopathy, or cytomegalovirus infection. Peripheral blood samples were collected, and all infants were administered intramuscular injections of 10 μg of the HBV vaccine at 0, 1, and 6 months. Infants with serum HBsAb levels >100 mIU/mL at 7 months were classified as high responders (HRs). Infants with serum HBsAb levels<10 mIU/mL received additional intramuscular injections of 10μgof the HBV vaccine three times over a period of 3–6 months. Infants with serum HBsAb levels that remained at <10 mIU/mL were recruited as non-responders(NRs). Ultimately,601 infants including 302 NR participants and 299 HR participants were recruited to be a part of the first study.

The second population consisted of adults recruited from Chongqing Daping Hospital. A total of1,845participantswere initially recruited in 2012. All of these participants had received HBV vaccines within the last year and provided written informed consent for participation in the study. Demographic information, smoking history, vaccination history, chronic disease status, immunosuppressive disease status and medication lists were obtained using questionnaires. In this population, 95 participants with serum HBsAb levels <10 mIU/mL were classified as NRs, and there were 1,479 participants with serum HBsAb levels >100 mIU/mL. We chose the HR participants after taking gender, age, BMI, and smoking status into account. At first, we excluded participants whose age and BMI were significant departures from those of the NRs. We then excluded certain individuals to keep the proportions of gender and smoking status among the HRs similar to those among the NRs. Ultimately, 135 participants were classified as HRs.

### SNP selection and genotyping

The DTX1 gene is located in the 12q24.13region, spanning approximately 40.17 kb. A total of 24 SNPs (minor allele frequencies (MAFs)>5%) in the DTX1 gene have been identified in the HapMap Han Chinese population from Beijing; these SNPs are located in non-coding regions (see S1 Fig). We selected 10 tagSNPs using the HaploviewV4.2 software program (using two-marker haplotypes). Genomic DNA was extracted from frozen venous blood using the TIANamp Blood DNA Kit (TIANGEN Biotech, Beijing, China) according to the manufacturer’s instructions.

SNP genotyping was performed using an iMLDR technique developed by Genesky Biotechnologies Inc. (Shanghai, China). We applied the iMLDR technique to genotype the 10 SNP loci in one ligation reaction. A multiplex of PCR reactions was designed to amplify the SNP loci. The first PCR reaction, performed in a 20-μL total volume, contained 1X PCR buffer (Takara, Dalian, China), 3.0 mM Mg^2+^, 0.3 mM dNTPs, 1 U of Hot-Start Taq DNA polymerase (Takara,Dalian, China), 1 μLof primer mixture 1, and ~20 ng of genomic DNA. The second PCR reaction, also performed in a 20μL total volume, contained 1X GC Buffer I (Takara), 3.0 mM Mg^2+^, 0.3 mM dNTPs, 1 U of Hot-Start Taq DNA polymerase (Takara), 1 μL of primer mixture 2, and ~20 ng of genomic DNA. The primer information is presented in S1 Table.

The PCR program for both reactions was 95°C for 2 min, 11 cycles (94°C for 20 s, 65°C-0.5°C/cycle for 40 s, and 72°C for 1 min and 30 s), 24 cycles (94°C for 20 s, 59°C for 30 s, and 72°C for 1 min and 30 s), 72°C for 2 min, and hold at 4°C. The two PCR products were equally mixed and purified by digestion with 1 U of shrimp alkaline phosphatase at 37°C for 1 h and at 75°C for 15 min. The ligation reaction, performed in a 20-μL total volume, contained 1 X ligation buffer, 80 U of Taq DNA Ligase (NEB), 1 μL of labeling oligo mixture, 2 μL of probe mixture, and 5 μL of purified PCR product mixture. The oligo or probe information for these mixtures is presented in S1 Table. The ligation cycling program was 95°C for 2 min, 38 cycles (94°C for 1 min and 56°C for 4 min), and hold at 4°C. A total of 0.5 μL of ligation product was loaded into an ABI 3730xl, and the raw data were analyzed using the GeneMapper 4.1 software program. All of the primers, probes, and labeling oligos were designed by and ordered from Genesky Biotechnologies, Inc. (Shanghai, China).

### Statistical analyses

**Fig 1:**
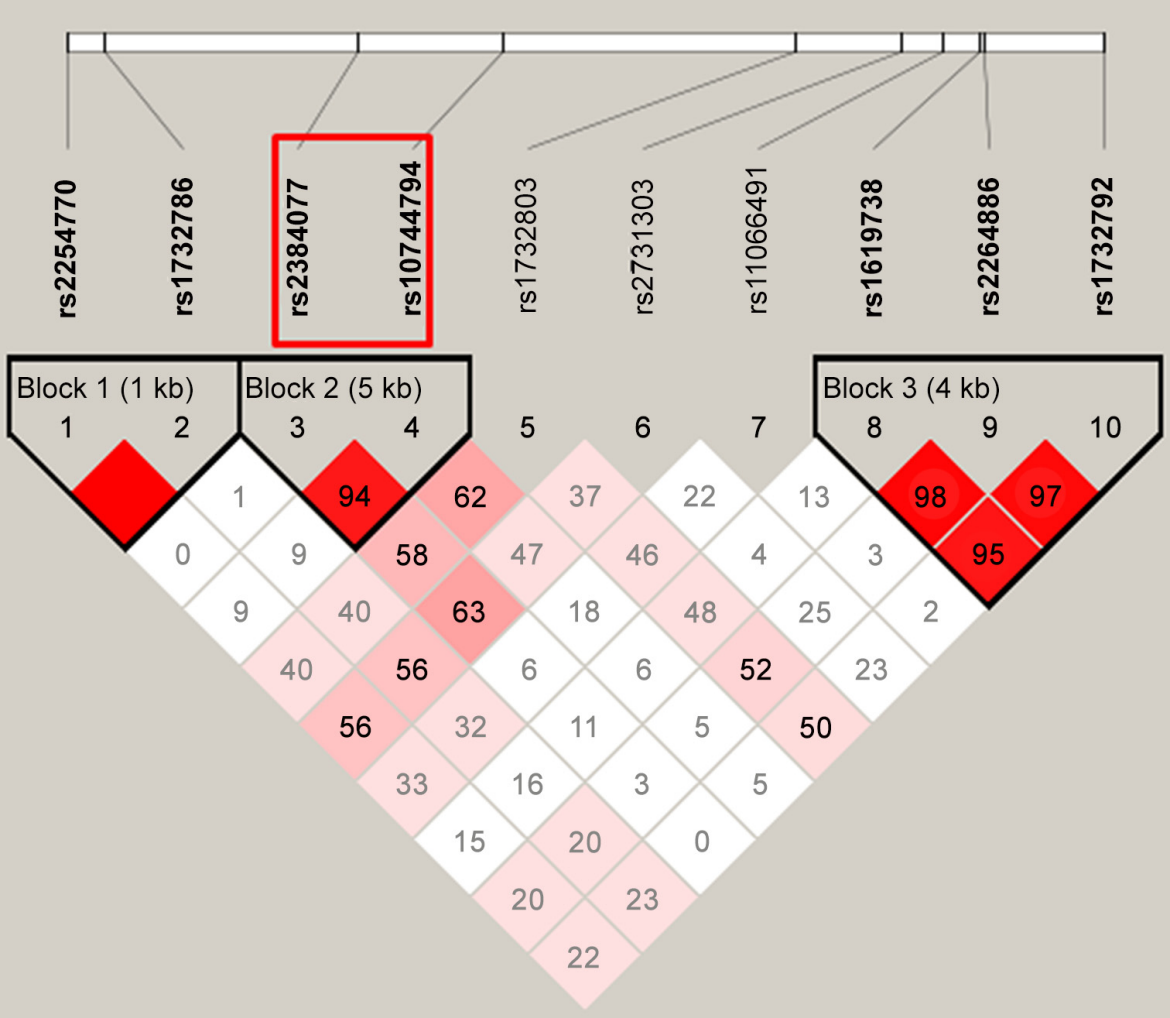
LD blocks of the 10 SNPs in DTX1.

SNP-specific deviations from the Hardy-Weinberg equilibrium (HWE) were tested using HWE exact tests (“genetics” package implemented in R language). Allele frequencies were compared between two groups using Pearson’s χ^2^ test, and genotype frequencies were compared between two groups using Fisher’s exact test. Bonferroni correction was used to correct the *p*-values. Logistic regression analysis was used to adjust for confounding factors. The association between genotyped polymorphisms and the risk of disease was estimated using corrected *p-*values, odds ratios (ORs), and 95% confidence intervals (CIs). Certain ORs of minor alleles that were less than 1 were converted into a value greater than or equal to 1.0 by calculating the ORs of the alternative alleles. The statistical analyses were performed using the PLINK 1.07 software program. All statistical tests were two sided, and corrected *p*-values<0.05 were considered to be statistically significant. The pairwise D measures of linkage disequilibrium (LD) for all SNPs as well as the haplotype distributions in the HRs and NRs were calculated using Haploview v4.2 software. A 95% CI for D’ between 0.7 and 0.98 was considered to indicate strong LD, and a block was created if 95% of the informative comparisons were considered to indicate “strong LD”. Haplotypes occurring with a frequency<1% were excluded. The 3 strongest LD blocks (with each block having 3 haplotypes) were then identified (see Fig 1). A haplotype association study was performed to test the set of blocks using Pearson’s χ^2^ test. Logistic regression analysis was used to adjust for confounding factors (age, gender, BMI and smoking status). Correction for multiple testing was performed separately in the infants, adults and combined data set using Bonferroni correction.

## Results

### Basic characteristics of the two study populations

The demographic and clinical characteristics of the two study populations are presented in Table 1. In the infant population, all of the infants were less than 6 years old, and no significant differences were noted in gender or BMI between the NR and the HR groups. In the adult population (1,845 healthy volunteers), 95 qualified as NRs (<10 mIU/mL), and 135 qualified as HRs (>100 mIU/mL). Moreover, no significant differences in gender or BMI were noted between the NR and the HR groups among the adults.

**Table 1:** Characteristics of the infant and adult populations.

Infants				Adults			
HBsAb	NR	HR	*p*	HBsAb	NR	HR	*p*
	N = 302	N = 299			N = 135	N = 95	
BMI (kg/m^2^)	17.51±3.67	17.68±4.12	0.071	BMI (kg/m^2^)	23.41±3.55	23.56±3.81	0.058
Age (months)	24.4±9.56	22.6±11.34	0.151	Age (years)	38.5±6.58	37.2±7.14	0.079
Gender				Gender			
Male	186	192	0.505	Male	82	53	0.453
Female	116	107		Female	53	42	
				Smoker			
				Yes	58	46	0.413
				No	77	49	

### Association between SNPs and immune response to hepatitis B vaccination

#### Infant population

In the genotyping experiments involving the 10 SNPs in the infant population, the call rate of all 10 SNPs was greater than 99%. More specifically, only one SNP (rs11066491) had a call rate below 100% (99.64%), so the remaining nine SNPs all had a call rate of 100% (S2 Table). HWE was analyzed for the infant population using PLINK 1.07, and all SNPs conformed to HWE (corrected *p*>0.05). All 601 samples had a call rate greater than 90%, and 600 of them had a call rate of 100%. Logistic regression analysis with an additive model was used to adjust for the confounding factors (age, gender and BMI). The allele and genotype distributions of the 10 tagSNPs in the NR and HR groups are presented in Table 2. Two SNPs (rs2384077 and rs10744794) were significantly associated with the immune response to the HBV vaccination (adjusted *p*<0.05). The SNPs rs2384077 and rs10744794 are both located in the first intron of DTX1. The frequency of the minor allele ‘G’ of rs2384077 was significantly reduced in the NR group relative to the HR group (adjusted *p* = 2.63E^-04^), with an adjusted OR of 1.9948 (95% CI: 1.4518–2.5974) for the alternative allele. The distribution of the genotypes significantly differed between the NR and the HR groups (adjusted *p* = 7.345E^-05^). Meanwhile, the frequency of the minor allele ‘C’ of rs10744794 was significantly reduced in the NR group compared with the HR group (adjusted *p* = 3.69E^-04^), and the adjusted OR was 1.6883 (95% CI: 1.2799–2.2232) for the alternative allele. The distribution of the genotypes between the NR and the HR groups was also significantly different for this allele (adjusted *p* = 5.23E^-05^).

**Table 2:** Minor allele and genotype frequencies of the SNPs associated with the response to hepatitis B vaccination in infants and adults.

			Minor allele					Genotype			
SNP	A1	A2	NR	HR	Adjusted *p*^a^(χ2 test)	Corrected *p*^b^	Adjusted OR (95% CI)^a^	NR	HR	Adjusted *p*^a^(Fisher’s exact test)	Corrected *p*^b^
**Infants**											
rs2254770	C	G	214	212	0.9954	1	1.0021(0.7931–1.269)	33/148/121	45/122/132	0.1134	1
rs1732786	G	A	215	215	0.9113	1	1.0063(0.7994–1.2845)^c^	33/149/120	45/125/129	0.1478	1
rs2384077	G	A	61	113	**2.63E-04**	**2.63E-03**	1.9948(1.4518–2.5974)^c^	4/53/245	5/103/191	**7.35E-05**	**7.35E-04**
rs10744794	C	T	97	148	**3.69E-04**	**3.69E-03**	1.6883(1.2799–2.2232)^c^	9/79/214	7/134/158	**5.23E-05**	**5.23E-04**
rs1732803	G	T	158	170	0.4242	1	1.1091(0.8628–1.4312)^c^	19/120/163	30/110/159	0.2874	1
rs2731303	A	C	124	146	0.1453	1	1.2268(0.9488–1.6311)^c^	13/98/191	13/120/166	0.18234	1
rs11066491	G	C	126	126	0.934	1	1.0097(0.757–1.3021)^c^	11/104/187	17/92/190	0.3936	1
rs1619738	G	C	149	144	0.8221	1	1.011(0.7919–1.341)	14/121/167	18/108/173	0.5234	1
rs2264886	G	A	121	116	0.7943	1	1.025(0.7821–1.377)	11/99/192	13/90/196	0.7885	1
rs1732792	A	G	133	123	0.5956	1	1.071(0.8263–1.421)	12/109/181	12/99/188	0.7793	1
**Adults**											
rs2254770	C	G	67	98	0.8667	1	1.0121(0.7138–1.5244)^c^	10/47/38	23/52/60	0.2034	1
rs1732786	G	A	68	99	0.8942	1	1.0298(0.6789–1.5179)^c^	10/48/37	23/53/59	0.1934	1
rs2384077	G	A	21	61	**2.16E-03**	**2.16E-02**	2.0268(1.3016–3.87)^c^	1/19/75	5/51/79	**4.56E-03**	**4.56E-02**
rs10744794	C	T	33	78	**4.82E-03**	**4.82E-02**	1.6852(1.121–2.7925)^c^	3/27/65	6/66/63	**4.26E-03**	**4.26E-02**
rs1732803	G	T	50	86	0.3216	1	1.3087(0.8666–1.9759)^c^	5/40/50	19/48/68	0.09745	0.9745
rs2731303	A	C	46	69	0.7815	1	1.0678(0.6892–1.6361)^c^	3/40/52	6/57/72	0.9683	1
rs11066491	G	C	41	60	0.8734	1	1.0316(0.6575–1.6121)^c^	4/33/58	9/42/84	0.0754	0.754
rs1619738	G	C	49	57	0.2499	1	1.201(0.8367–1.989)	6/37/52	8/41/86	0.3843	1
rs2264886	G	A	38	45	0.3673	1	1.18(0.7633–1.974)	5/28/62	6/33/96	0.6634	1
rs1732792	A	G	41	47	0.2735	1	1.194(0.8061–1.995)	5/31/59	5/37/93	0.5782	1
**Combined**											
rs2254770	C	G	281	310	0.8992	1	1.0009(0.823–1.2309)^c^	43/195/159	68/174/192	0.02013	0.2013
rs1732786	G	A	283	314	0.8341	1	1.0118(0.834–1.2452)^c^	43/197/157	68/178/188	0.0232	0.232
rs2384077	G	A	82	174	**1.17E-05**	**1.17E-04**	2.0412(1.4892–2.586)^c^	5/72/320	10/154/270	**3.57E-07**	**3.57E-06**
rs10744794	C	T	130	226	**3.93E-05**	**3.93E-04**	1.6658(1.3086–2.0433)^c^	12/106/279	13/200/221	**4.46E-07**	**4.46E-06**
rs1732803	G	T	208	256	0.1547	1	1.1438(0.8993–1.4484)^c^	24/160/213	49/158/227	0.02657	0.2657
rs2731303	A	C	170	215	0.2101	1	1.1678(0.905–1.4903)^c^	16/138/243	19/177/238	0.1834	1
rs11066491	G	C	167	186	0.9074	1	1.0147(0.8032–1.2768)^c^	15/137/245	26/134/274	0.3721	1
rs1619738	G	C	198	201	0.4526	1	1.0796(0.8806–1.388)	20/158/219	26/149/259	0.2874	1
rs2264886	G	A	159	161	0.4847	1	1.085(0.8558–1.3762)	16/127/254	19/123/292	0.6107	1
rs1732792	A	G	174	170	0.3114	1	1.113(0.8793–1.415)	17/140/240	17/136/281	0.5035	1

a: *p*-values and OR (95% CI) adjusted for age, gender, BMI, and smoking status (for adults and combined) using logistic regression.

b: *p*-values corrected by Bonferroni correction.

c: adjusted ORs for the alternative alleles of the minor alleles (to keep the OR greater than or equal to 1).

#### Adult population

In the adult population, the call rates of the SNPs and samples were greater than 95%, and all SNPs conformed to HWE. Logistic regression analysis with an additive model was used to adjust for the confounding factors (age, gender, BMI and smoking status). The allele and genotype distributions of the adult population are presented in Table 2. The same two SNPs (rs2384077 and rs10744794) were significantly associated with the immune response to the HBV vaccination. The frequency of the minor allele ‘G’ of rs2384077 was significantly reduced in the NR group compared with the HR group (adjusted *p* = 2.16E^-03^), and the adjusted OR was 2.0268 (95% CI: 1.3016–3.87) for the alternative allele. The distribution of the genotypes between the NR and the HR groups was significantly different (adjusted *p* = 4.56E^-03^). Meanwhile, the frequency of the minor allele ‘C’ of rs10744794 was significantly reduced in the NR group compared with the HR group (adjusted *p* = 4.82E^-03^), with an adjusted OR of 1.6852 (95% CI: 1.121–2.7925) for the alternative allele. The distribution of the genotypes between the NR and the HR groups was also significantly different for this allele (adjusted *p* = 4.26E^-03^).

#### Combined analyses

We also combined the two populations for a genotyping experiment. Logistic regression analysis with an additive model was used to adjust for the confounding factors (age, gender, BMI and smoking status). The same two SNPs were both highly significantly associated with the immune response to the HBV vaccination. The adjusted *p-*values forrs2384077 and rs10744794 were 1.17E^-05^ and 3.93E^-05^, respectively, and the adjusted ORs were 2.0412 (95%CI: 1.4892–2.586) and 1.6658 (95%CI: 1.3086–2.0433) for the respective alternative alleles. The distribution of the genotypes between the NR and the HR groups was also significantly different (adjusted *p* = 3.57E^-07^ and 4.46E^-07^, respectively).

### Haplotype analysis and functional element annotation for two SNPs

To assess the functional relevance of these two SNPs, we constructed haplotypes for 10 SNPs using the accelerated Expectation Maximization algorithm, implemented usingHaploviewV4.2 software. As presented in Fig 1, rs2384077 was in LD with rs10744794 as a block (block 2, r^2^ = 0.94). As shown in Table 3, the haplotypes of the LD block were significantly associated with the immune response to the HBV vaccination in each population and in the combined results. The significant individual haplotypes included A-T (infants: adjusted *p* = 4.14E^-05^, adults: adjusted *p* = 2.03E^-02^, combined: adjusted *p* = 3.67E^-06^) and G-C (infants: adjusted *p* = 6.24E^-03^, adults: adjusted *p* = 4.10E^-03^, combined: adjusted *p* = 5.33E^-06^). The other two LD blocks were not significantly associated with the vaccine-induced immune response (data not shown).

**Table 3:** Haplotype effects for DTX1 (rs2384077-rs10744794).

Haplotype	Frequency	Case, Control Frequencies	*p*(χ2 test)	Adjusted *p^a^*	Corrected *p^b^*
**Infants**					
AT	0.79	0.839, 0.740	**2.58E-05**	**4.14E-05**	**3.73E-04**
GC	0.138	0.101, 0.177	**1.00E-04**	**6.24E-03**	0.0562
AC	0.065	0.060, 0.071	0.4436	0.2564	1
**Adults**					
AT	0.747	0.826, 0.691	**0.0011**	**2.30E-02**	0.207
GC	0.166	0.110, 0.206	**0.0063**	**4.10E-03**	**0.0369**
AC	0.075	0.064, 0.083	0.4489	0.5722	1
**Combined**					
AT	0.778	0.836, 0.725	**5.62E-08**	**3.67E-06**	**3.30E-05**
GC	0.146	0.103, 0.186	**1.72E-06**	**5.33E-06**	**4.80E-05**
AC	0.068	0.061, 0.074	0.2697	0.5632	1

a: *p*-values adjusted for age, gender, BMI, and smoking status (adults) using logistic regression.

b: *p*-values corrected by Bonferroni correction.

**Fig 2:**
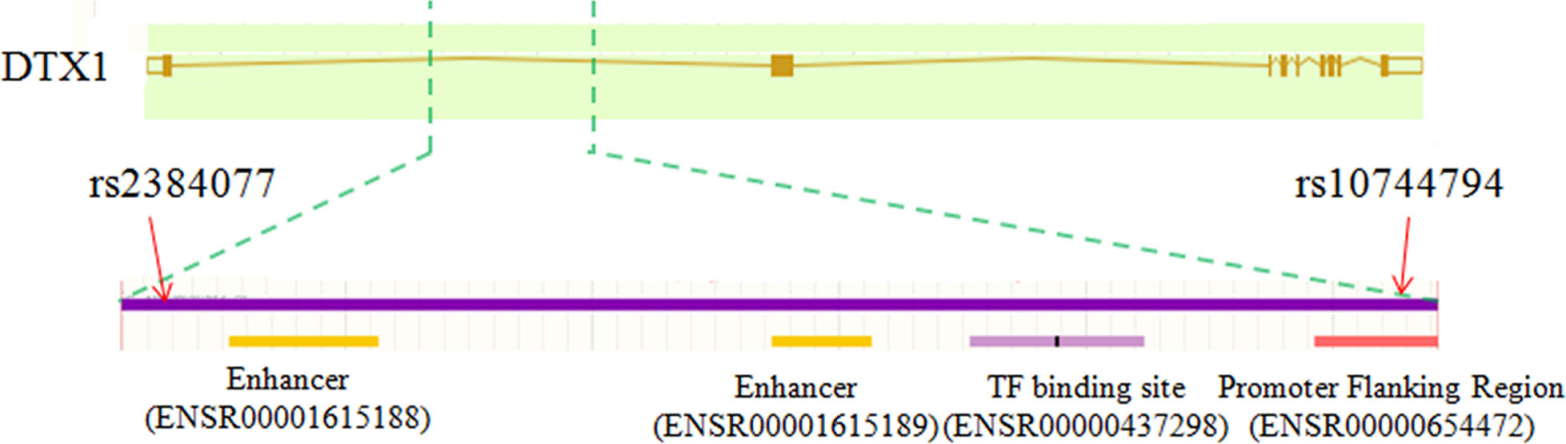
Regulatory elements in the LD block of rs2384077 and rs10744794.

To further explore the functional mechanism by which the two SNPs in the 5-kb region (LD block) affect immunity following vaccination against HBV, we investigated this region by functional element annotation using the Regulatory Features of the Ensembl database (url: http://asia.ensembl.org/info/genome/funcgen/index.html). As presented in Fig 2, four transcriptional regulatory elements, including two enhancers (ENSR00001615188, ENSR00001615189), one TF-binding site (ENSR00000437298) and one promoter-flanking region (ENSR00000654472), were located in this region. rs10744794 is located in the promoter-flanking region (ENSR00000654472).

## Discussion

Immunity following HBV vaccination is affected by several different factors; however, the molecular mechanism underlying this response is unclear. Several studies have identified numerous immune response-related genes and SNPs that were significantly associated with the degree of the immune response to HBV vaccination, including HMC, cytokine, and cytokine receptor genes [26–28]. T cell immunity plays an important role in regulating the magnitude of the immune response to HBV vaccination [29, 30] because genes related to T cell immunity are involved in the host response to the HBV vaccination; however, there have been limited reports on polymorphisms in genes involved in T cell immunity.

DTX1 is a single transmembrane protein that is involved in T cell anergy [23, 24]. Deltex (DTX) contains a proline-rich motif and a RING finger domain at its C terminus and two WWE domains at its N terminus (the WWE domains mediate its interaction with Notch) [31]. Several studies have suggested that DTX plays a critical role in the recombination signal binding protein for the immunoglobulin kappa J region (RBP-J)-independent Notch signaling pathway [32–34]. DTX1 over-expression inhibits Notch signaling, as demonstrated by enhanced B cell lymphocyte development and suppressed T cell development [35, 36]. Evaluating the relationship between DTX1 and the immune response may shed further light on the immune response to the HBV vaccination.

In the present study, we aimed to evaluate how DTX1 might be involved in the host response to the HBV vaccination in the Chinese Han population. We detected 10 SNPs in DTX1 using iMLDR. Two of these SNPs, located in the first intron of DTX1 (rs2384077 and rs10744794),were significantly associated with the degree of the immune response to HBV vaccination. Introns comprise a large portion of the eukaryotic genome, and as such, these introns contain abundant biological information [37]. Numerous studies have demonstrated that introns participate in various biological processes, such as regulating transcription initiation, mRNA modification, metabolism, transport, and protein expression [38–40]. Although various studies have found that the pathogenic risk of SNPs in intron regions is significantly reduced compared with the risk of SNPs in the gene coding and regulatory regions, the pathogenic risk of SNPs in the first intron is typically increased [41]. Therefore, to explore the functional relevance of the two identified SNPs, we constructed their haplotypes. The results demonstrated that the haplotypes were significantly associated with the immune response to HBV vaccination in both infant and adult populations.

Subsequently, to further explore the functional mechanism of the LD block (5-kb region) of the two SNPs in the immune response to vaccination against HBV, functional element annotation using the Regulatory Features of the Ensembl database was performed. We identified four transcriptional regulatory elements, including two enhancers (ENSR00001615188, ENSR00001615189), one TF-binding site (ENSR00000437298), and one promoter-flanking region (ENSR00000654472), located in this region, with rs10744794 being located in the promoter-flanking region (ENSR00000654472). These results indicate that the SNPs in this LD block (5-kb region) may affect DTX1 expression by altering the four transcriptional regulatory elements and T cell immunity.

The induction of immunity following HBV vaccination is a complicated process affected by several factors, including environmental and host-related physical factors, such as age, smoking, and alcohol consumption. In the present study, we identified two DTX1 SNPs that are involved in the host response to the HBV vaccination using peripheral blood samples from an infant population, which allowed us to avoid interference by most exogenous factors. In a second population, we used peripheral blood samples from adults to confirm these results. In the adult population, we took age, BMI, and smoking status into account. The results from both populations demonstrated that the two identified SNPs were significantly associated with the degree of the immune response to HBV vaccination, indicating that the results of the association analysis in our study were efficient and reliable.

There are still certain limitations to our study. First, a lack of power caused by the moderate sample size in our study prevented us from assessing genetic effects in NRs to the HBV vaccine. Second, although we only chose ethnic Han for our study, there are many other ethnic populations in the Southwest than in other places in China, which might have introduced heterogeneity in our Han population. Third, we could not collect information on the doses and sources of the vaccine that was used in the adult cohort, which might have biased the outcome of the statistical analysis. Moreover, due to a lack of enough blood samples, we could not detect the expression level of DTX1 in T cells to confirm a relationship between DTX1 expression and the two identified SNPs. Therefore, we will collect more samples for further study, and greater attention will be paid to investigating the relationship between DTX1 expression and the two identified SNPs.

In summary, the results of the present study demonstrate that the minor allele ‘G’ of rs2384077 and the minor allele ‘C’ of rs10744794 in the first intron of the DTX1 gene are associated with a higher immune response to the HBV vaccination in the Chinese Han population in Southwest China. The haplotype analysis also indicated that the LD block of the two SNPs is significantly associated with the immune response to HBV vaccination. Functional element annotation of the LD block between the two SNPs found four transcriptional regulatory elements located in this 5-kb region. Considering all of these results, we speculated that the two SNPs are involved in the immunity generated by vaccination against HBV, likely by affecting DTX1 expression. Nevertheless, the relationship between DTX1 expression and the two SNPs needs further confirmatory research. Additional studies that focus on the molecular mechanism(s) underlying how DTX1 affects the immune response to vaccination against HBV might lead to a better understanding of the mechanism of action of these vaccines.

## Supporting Information

S1 Fig LD plot for the 24 SNPs (MAFs>5%). (TIF)

S1 Table Primer and oligo or probe information for the 10 SNPs detected by iMLDR. (XLSX)

S2 Table Statistical information regarding the 10 SNPs. (XLSX)
