# Supplementary figures and images for: Deltex1 Polymorphisms Are Associated with Hepatitis B Vaccination Non-Response in Southwest China
Source: PLoS One. 2016 Feb 19;11(2):e0149199. doi: 10.1371/journal.pone.0149199 (PMC4760674; doi:10.1371/journal.pone.0149199)

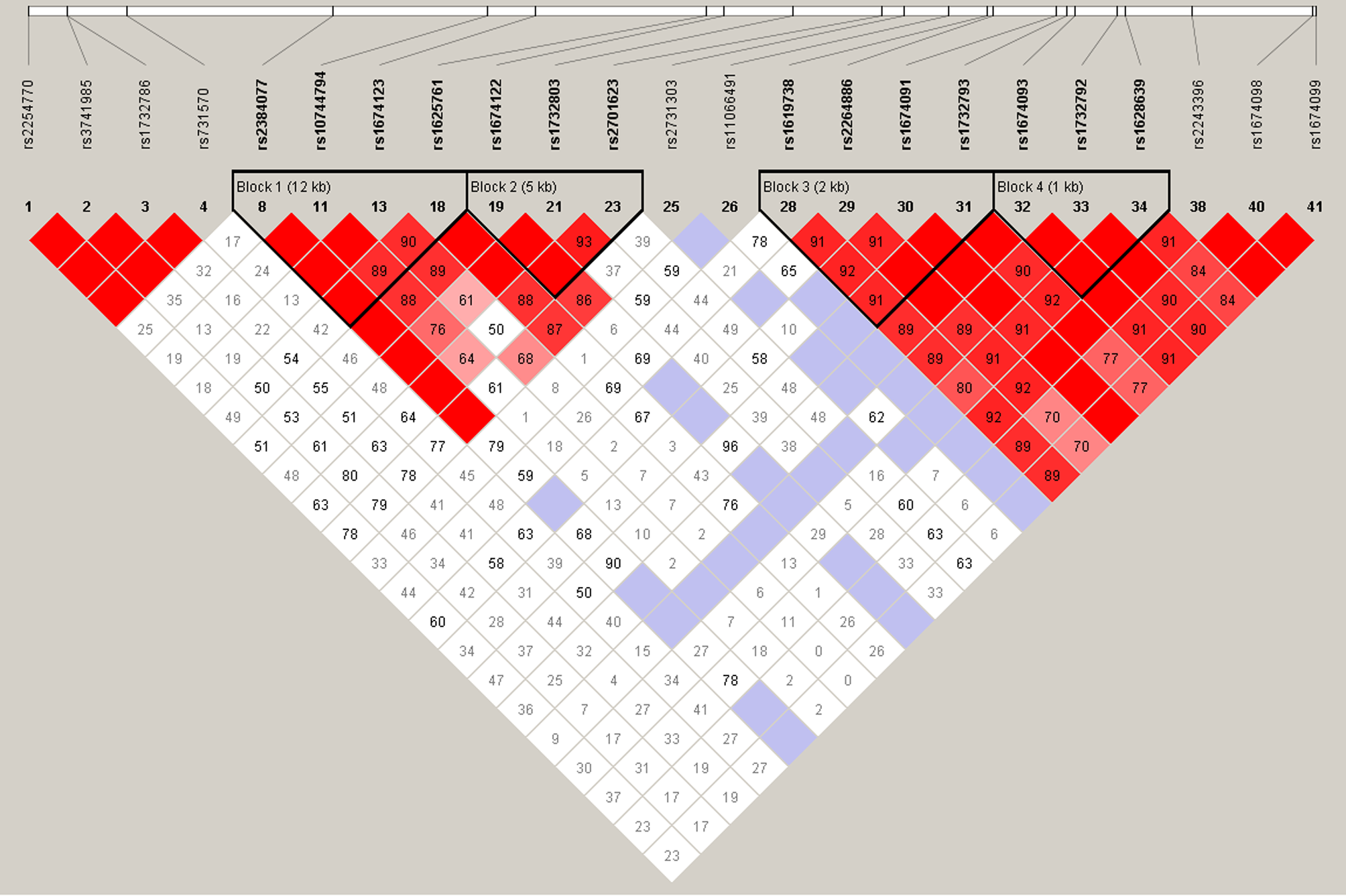

Supplement: S1 Fig — (TIF) [file pone.0149199.s001.tif]
